# Respiratory Nasal Mucosa in Chronic Rhinosinusitis with Nasal Polyps versus COVID-19: Histopathology, Electron Microscopy Analysis and Assessing of Tissue Interleukin-33

**DOI:** 10.3390/jcm10184110

**Published:** 2021-09-12

**Authors:** Ionuț Isaia Jeican, Dan Gheban, Lucian Barbu-Tudoran, Patricia Inișca, Camelia Albu, Maria Ilieș, Silviu Albu, Mihaela Laura Vică, Horea Vladi Matei, Septimiu Tripon, Mihaela Lazăr, Maria Aluaș, Costel Vasile Siserman, Monica Muntean, Veronica Trombitas, Cristina Adela Iuga, Iulian Opincariu, Lia Monica Junie

**Affiliations:** 1 Department of Head and Neck Surgery and Otorhinolaryngology, University Clinical Hospital of Railway Company, Iuliu Hatieganu University of Medicine and Pharmacy, 400015 Cluj-Napoca, Romania; jeican.ionut@umfcluj.ro (I.I.J.); veronicatrombitas@gmail.com (V.T.); 2 Department of Anatomy and Embryology, Iuliu Hatieganu University of Medicine and Pharmacy, 400006 Cluj-Napoca, Romania; iulian.opincariu@umfcluj.ro; 3 Department of Pathology, Iuliu Hatieganu University of Medicine and Pharmacy, 400015 Cluj-Napoca, Romania; Curta.Camelia@umcluj.ro; 4 Electron Microscopy Laboratory, Faculty of Biology and Geology, Babes-Bolyai University, 400006 Cluj-Napoca, Romania; lucian.barbu@ubbcluj.ro (L.B.-T.); tripon_septimiu@yahoo.com (S.T.); 5 Electron Microscopy Integrated Laboratory, National Institute for R&D of Isotopic and Molecular Technologies, 400293 Cluj-Napoca, Romania; 6 Department of Pathology, County Emergency Hospital, 330084 Deva, Romania; patricia.bilei@gmail.com; 7 Imogen Medical Research Institute, County Clinical Emergency Hospital, 400014 Cluj-Napoca, Romania; 8 Department of Proteomics and Metabolomics, MedFuture Research Center for Advanced Medicine, Iuliu Hatieganu University of Medicine and Pharmacy, 400349 Cluj-Napoca, Romania; ilies.maria@umfcluj.ro (M.I.); iugac@umfcluj.ro (C.A.I.); 9 Department of Cell and Molecular Biology, Iuliu Hatieganu University of Medicine and Pharmacy, 400349 Cluj-Napoca, Romania; mvica@umfcluj.ro (M.L.V.); hmatei@umfcluj.ro (H.V.M.); 10 Institute of Legal Medicine, 400006 Cluj-Napoca, Romania; csiserman@umfcluj.ro; 11 Cantacuzino National Military-Medical Institute for Research and Development, 050096 Bucharest, Romania; lazar.mihaela@cantacuzino.ro; 12 Department of Oral Health, Iuliu Hatieganu University of Medicine and Pharmacy, 400012 Cluj-Napoca, Romania; 13 Department of Legal Medicine, Iuliu Hatieganu University of Medicine and Pharmacy, 400015 Cluj-Napoca, Romania; 14 Department of Infectious Disease, Clinical Hospital of Infectious Disease, Iuliu Hatieganu University of Medicine and Pharmacy, 400000 Cluj-Napoca, Romania; monica.muntean@umfcluj.ro; 15 Department of Pharmaceutical Analysis, Faculty of Pharmacy, Iuliu Hatieganu University of Medicine and Pharmacy, 400349 Cluj-Napoca, Romania; 16 Department of Microbiology, Iuliu Hatieganu University of Medicine and Pharmacy, 400349 Cluj-Napoca, Romania; mjunie@umfcluj.ro

**Keywords:** nasal mucosa, chronic rhinosinusitis, nasal polyps, COVID-19, interleukin-33

## Abstract

(1) Background: Chronic rhinosinusitis with nasal polyps (CRSwNP) is one of the most studied rhinological disorders. Modifications of the respiratory nasal mucosa in COVID-19 patients are so far unknown. This paper presents a comparative morphological characterization of the respiratory nasal mucosa in CRSwNP versus COVID-19 and tissue interleukin (IL)-33 concentration. (2) Methods: We analyzed CRSwNP and COVID-19 samples through histopathology, scanning and transmission electron microscopy and performed proteomic determination of IL-33. (3) Results: Histopathologically, stromal edema (*p* < 0.0001) and basal membrane thickening (*p* = 0.0768) were found more frequently in CRSwNP than in COVID-19. Inflammatory infiltrate was mainly eosinophil-dominant in CRSwNP and lymphocyte-dominant in COVID-19 (*p* = 0.3666). A viral cytopathic effect was identified in COVID-19. Scanning electron microscopy detected biofilms only in CRSwNP, while most COVID-19 samples showed microbial aggregates (*p* = 0.0148) and immune cells (*p* = 0.1452). Transmission electron microscopy of CRSwNP samples identified biofilms, mucous cell hyperplasia (*p* = 0.0011), eosinophils, fibrocytes, mastocytes, and collagen fibers. Extracellular suggestive structures for SARS-CoV-2 and multiple Golgi apparatus in epithelial cells were detected in COVID-19 samples. The tissue IL-33 concentration in CRSwNP (210.0 pg/7 μg total protein) was higher than in COVID-19 (52.77 pg/7 μg total protein) (*p* < 0.0001), also suggesting a different inflammatory pattern. (4) Conclusions: The inflammatory pattern is different in each of these disorders. Results suggested the presence of nasal dysbiosis in both conditions, which could be a determining factor in CRSwNP and a secondary factor in COVID-19.

## 1. Introduction

The respiratory nasal mucosa represents an important component of immunity, both as a barrier against pathogenic respiratory agents, allergens, and physical insults, and maintains the in homeostasis between commensal microbiota and pathogenic agents inhaled through the nose [1,2]. The respiratory nasal mucosa mediates local and systemic inflammatory responses to a wide range of pathogens. Epithelium-derived cytokines are important regulatory factors in inflammatory responses and in linking innate and adaptive immunity [3].

Chronic rhinosinusitis (CRS) with nasal polyps (CRSwNP) is a complex type 2 inflammatory disease of the respiratory nasal mucosa, which is frequently found and has a multidirectional impact on quality of life [4]. The study of the nasal mucosa in CRSwNP remains of interest because for some patients standard medical and surgical therapies do not provide sufficient control of inflammation (refractory disease). Structured histopathology, effector cells, and cytokines involved in the pathogenesis of CRSwNP are a growing field of interest related to targeted immunomodulatory pharmacotherapy. Biologic therapies target interleukin IL-4Rα, IL-5, IL-5Rα, IL-33, immunoglobulin E, and thymic stromal lymphopoietin [5].

The novel RNA beta-coronavirus, Severe Acute Respiratory Syndrome-CoronaVirus-2 (SARS-CoV-2), is the etiological agent for COronaVIrus Disease 19 (COVID-19) [6], a respiratory infection with early nasal pathogenesis that can extend to systemic damage. Nasal cells are the site of the first step of infection: these cells express the highest levels of angiotensin-converting enzyme 2 (ACE2) and of the cellular serine protease TMPRSS2, the main entry receptors for SARS-CoV-2 [1,7]. The histopathological and ultramicroscopic alterations induced by SARS-CoV-2 on the respiratory nasal mucosa are unknown.

Several biomarkers have been studied in CRSwNP such as cytokines (IL-4, -5, -13, -33, TNFα, LT4) [8]. IL-33 is a tissue-derived nuclear cytokine from the IL-1 family, abundantly expressed in epithelial cells during homeostasis and inflammation. IL-33 is an essential immune modulator in type 2 immune responses involved in chronic allergic, fibrotic, infectious, and inflammatory diseases [9,10]. IL-33 plays an important role in the pathogenesis of CRSwNP. Kim et al. [11] found in 69 CRSwNP patients higher protein levels of IL-33 in uncinate process tissues (median: 0.917 ng/mg) than in controls (medians: 0.187 ng/mg), *p* < 0.001. Also, it seems that IL-33 production is also linked to SARS-CoV-2 infection, the serum concentration being correlated with prognosis [12,13,14].

Thus, we aimed to perform a histopathological and ultramicroscopic comparative characterization of the respiratory nasal mucosa of patients with CRSwNP and those with COVID-19. Furthermore, the different tissue concentrations of IL-33 in the nasal mucosa of CRSwNP and COVID-19 patients were assessed. The study compared severe disease in both situations: CRS patients in an advanced stage, where surgery was necessary, and COVID-19 patients who suffered a fatal outcome.

## 2. Materials and Methods

### 2.1. Study Design and Population

In two distinct prospective studies carried out in different periods, we assessed the histopathology, electron microscopy images, and tissue IL-33: a CRSwNP study and a COVID-19 study. The research design, the inclusion and exclusion criteria are presented in Table 1.

In addition, medical data on clinical information, demographics, comorbid conditions, and results of laboratory tests were collected.

The *CRSwNP study* was conducted in accordance with the guidelines of the Declaration of Helsinki, and the written informed consent of patients was obtained a day before surgery. Patients agreed to the collection of mucosal samples during surgery and to the sample investigation: histopathology, electron microscopy analysis and IL-33 quantitative analysis. Access to patients’ files and personal data such as samples was allowed only to the research team to respect patient confidentiality and privacy. The harvesting protocol for this study was approved by the Iuliu Hatieganu University of Medicine and Pharmacy Ethics Committee under No. 87/2018 and No. 388/2020.

For the *COVID-19 study*, an informed consent statement for autopsy was not required; autopsy is mandatory under Romanian Law (Law 104/2003 on handling bodies and the removal of organs and tissues for the purpose of transplantation; Government Decision no. 451/2004 on methodological norms for the application of law 104/2003; Law 271/2004 related to organizing forensic medicine activities in Romania; Procedure norms of forensic medicine activities, Ministry of Justice Order no. 1134/C/2000, and Health Ministry Order no. 255/2000). According to the Romanian legal framework, pathological autopsy must be performed on all patients deceased in the hospital if it is necessary to confirm, specify, or complete the clinical diagnosis or for forensic diagnosis.

Sino-nasal mucosa samples were removed from deceased COVID-19 patients while complying with international and national recommendations [15,16,17,18]. The harvesting protocol for the study was approved by the County Emergency Hospital Deva Ethics Committee under No. 8942/2021, by the Administrative Department of the County Emergency Hospital Deva under No. 8943/2021, and by the Administrative Department of the Institute of Legal Medicine Cluj-Napoca, under No. 4354/XII/615/2021.

### 2.2. Sampling

*CRSwNP study*. After induction of general anesthesia by oro-tracheal intubation, the nasal vestibule was cleaned with iodine, the nasal cavity was washed with saline solution, and a local vasoconstricting agent was administered. During FESS, five mucosal samples were obtained from the ethmoid bulla of each patient: one for histopathology, stored in formaldehyde 7%; two for electron microscopy (scanning and transmission), stored in glutaraldehyde 2.7%; and one for IL-33 quantitative analysis.

*COVID-19 study*. Five mucosal samples from the ethmoid bulla were obtained 12 h after death, by curettage with a Volkmann curette no. 2, using appropriate protective measures: one for real-time PCR (RT-PCR) SARS-CoV-2, and the other four fragments were distributed for analysis in the same way as mentioned above.

The samples for RT-PCR SARS-CoV-2 were stored in a viral transport medium (BioSci virus sampling tube model FBY, Darkewe Biotech Co. Ltd., Shenzhen, China). The tubes were stored immediately after collection in a freezer at a temperature of −20 °C; then, they were transferred to a freezer at a temperature of −80 °C over the next 24 h and stored until analysis.

The samples for electron microscopy were sent to the electron microscopy laboratory for processing immediately after collection. The samples for determining IL-33 were stored immediately after collection at −80 °C until analysis.

### 2.3. Real-Time PCR (RT-PCR) SARS-CoV-2 (for the COVID-19 Study)

For RT-PCR SARS-CoV-2 testing of mucosal tissue samples, total RNA isolation was performed with EPICENTRE MasterPure^TM^ Complete DNA and RNA Purification Kit (Illumina Company, Madison, WI, USA) according to the manufacturer’s instructions. The RNA samples were amplified on a QuantStudio™ 5 Real-Time PCR System (Thermo Fisher Scientific Inc., Waltham, MA, USA) using Logix Smart Coronavirus Disease 2019 (COVID-19) Kit (Co-Diagnostics Inc., Salt Lake City, UT, USA). The RdRp gene assay (RNA-dependent RNA polymerase gene—inside the Orf1ab polyprotein gene) was used as the target gene. The assay also included RNase P target as an internal positive control (IC) and a positive control that included 2 synthetic RNA molecules carrying sequences that were homologous to the RdRp of SARS-CoV-2 and were targeted by this assay (Ct 25.2). Co-primers targeting SARS-CoV-2 t-RNA were labeled with FAM fluorophore, and co-primers targeting positive internal DNA control were labeled with CAL Fluor Red 610 fluorophore. The following program was used: reverse transcription for 15 min at 45 °C, initial denaturation for 2 min at 95 °C, and 50 cycles of amplification (3 s at 95 °C and 32 s at 55 °C). The presence of a curve with quantification cycle (Cq) ≤ 45 cycles indicated a positive result.

### 2.4. Histopathology

The samples were fixed in formaldehyde 7% for 5 days, after which the samples were oriented and placed in cassettes. Tissue processing was performed using a vacuum infiltration processor, Tissue-Tek VIP 5 Jr (Sakura, Alphen aan den Rijn, The Netherlands). Paraffin embedding and sectioning were performed using the Tissue-Tek TEC 6 system (Sakura, Alphen aan den Rijn, The Netherlands) and Accu-Cut SRM 200 Rotary Microtome (Sakura, Alphen aan den Rijn, The Netherlands). Slide staining was performed using the automated slide stainer Tissue-Tek Prisma Plus (Sakura, Alphen aan den Rijn, The Netherlands) according to the internal staining protocol, using Mayer Modified Hematoxylin (Titolchimica, Rovigo, Italy) and Eosin solution (10 g Eosin B in 1000 mL distilled water). For Gram staining, the Gram Stain Kit (Gram Fuchsin Counterstain) (Atom Scientific, Manchester, UK) was used according to the manufacturer’s instructions.

Microscopic examination was performed by the same experienced pathologist (D.G.), using an Olympus BX46 clinical microscope (Olympus Europe SE & Co, Hamburg, Germany) with dedicated image acquisition camera and software. All sections were examined at 400× magnification.

### 2.5. Scanning Electron Microscopy (SEM)

The samples were fixed in glutaraldehyde 2.7% for 2 h, washed with phosphate buffered saline (PBS) and then with distilled water, then left to dry. The dried samples were glued to a support with silver paste and sputter coated with a 10 nm thick gold layer before imaging (Agar Auto Sputter Coater, Agar Scientific Ltd., Stansted, Essex, UK). Scanning electron microscopy (SEM) was conducted on a Hitachi SU8230 cold field emission gun (Tokyo, Japan) at 30 kV.

The aspect of the mucosa, microbial presence, biofilm identity, and ciliary patterns were investigated. Bacterial biofilm positivity was defined according to the diagnostic criteria of Mladina et al. [19]. Microbial aggregates were groups of more than 5 microorganisms placed next to one another, adhering to the surface of the mucosa but unenclosed in an extracellular matrix. All samples were examined by the same experienced investigator (L.B.T.).

### 2.6. Transmission Electron Microscopy (TEM)

The tissues fixed in glutaraldehyde (2.7% in 0.1 M PBS) for 120 min were rinsed 3 times with 0.15 M PBS for 1 h each and postfixed in 2% osmium tetroxide. Dehydration was accomplished with a series of mixtures (acetone 30, 50, 70, 80, 90, three times 100%). Inclusion was made with Epon 812 (EMS USA, Electron microscopy Sciences). The dehydrated tissue was then placed in a polymerization mixture according to the manufacturer’s protocol and left overnight at room temperature for final mixing and embedding. Polymerization was performed with a freshly prepared mixture of the above composition for 2 days at 55 °C. Ultrathin sections, about 90 nm thick, were obtained using a Leica-UC7 ultramicrotome and a diamond knife (Diateome, Swiss) (Leica Microsystems, Bensheim, Austria). The sections were collected on copper grids covered by a thin layer of Formvar. Final staining of the sections included treatment with Uranyless (Agar Scientific, Stansted, UK) for 2 min and with lead citrate for 2 min. Transmission electron microscopy (TEM) was conducted on a Jeol 1010 cold field emission gun (Tokyo, Japan).

The aspects of epithelial cells, mucosal cells, and microbial presence were investigated. All samples were examined by the same experienced investigator (L.B.T.).

Differences among the groups were analyzed with Fisher’s exact test and a chi-square test with the confidence interval set at 95%, and *p* < 0.05 was considered statistically significant (GraphPad Prism 5.03).

### 2.7. Assessing Tissue IL-33

#### 2.7.1. Tissue Lysate Preparation and Protein Extraction

Nasal mucosal tissues were rinsed with PBS, and protein extraction was performed in urea/thiourea solution (8/2 M, VWR, Lutterworth, UK) with cryogenic bead mill extraction (25 1/s, 3 min, 3 mm diameter balls) (Mixer Mill 400, Retsch, Hann. Münden, Germany) and subsequent applications of 6 cycles of sonication (5 × 3 s pulses, 19 kHz, 80% amplitude) (Ultrasonic bath, Bandelin, Germany). Next, the samples were centrifuged (1 h, 4 °C, 5430/5430R) and the supernatant was further subjected to total protein concentration determination by Bradford using bovine serum albumin as standard.

#### 2.7.2. Quantitative Analysis of IL-33 by ELISA

Tissue lysate levels of human IL-33 were assessed using sandwich enzyme-linked immunosorbent assays (ELISA). Individual tissue lysate samples containing 7 µg total protein each were measured in duplicate with the IL-33 ELISA kit (R&D Systems, Minneapolis, MN, USA, catalog number D3300B, sensitivity 0.069–1.510 pg/mL, mean minimum detectable dose 0.357 pg/mL, intra-assay precision CV = 3.7–5.9% and inter-assay precision CV = 4.4–6.0%) following the manufacturer’s instructions. A calibration curve was generated using the protein standard included into the kit. Absorbance was measured with a ClarioStarplate reader (BMG Labtech, Ortenberg, Germany), and data were acquired and processed with integrated Mars software. For quantification, a 4-parameter fit calibration curve was used, and final concentrations were calculated as the mean of two measurements (pg/7 µg total protein content).

Outliers were tested with Grubb’s test by setting the significance level as standard (alpha = 0.05); no significant outliers were identified in the data set. Data were presented as mean ± standard error of the mean. Differences among the groups were analyzed by employing the unpaired *t* test with Welch’s correction, and for data visualization, box plots were used (GraphPad Prism, San Diego, CA, USA).

## 3. Results

The distribution of the patients by age and studies, as well as the results obtained, is illustrated in Table 2.

**RT-PCR**. For the COVID-19 group, the median Ct value of the positive mucosal samples was 24 for the *RdRp* gene (IQR, 10–35). Genomic load (Ct ≤ 25) was high in 58.3% (*n* = 7/12) and intermediate (25 < Ct < 35) in 41.7% of the samples (*n* = 5/12).

**Figure 1:**
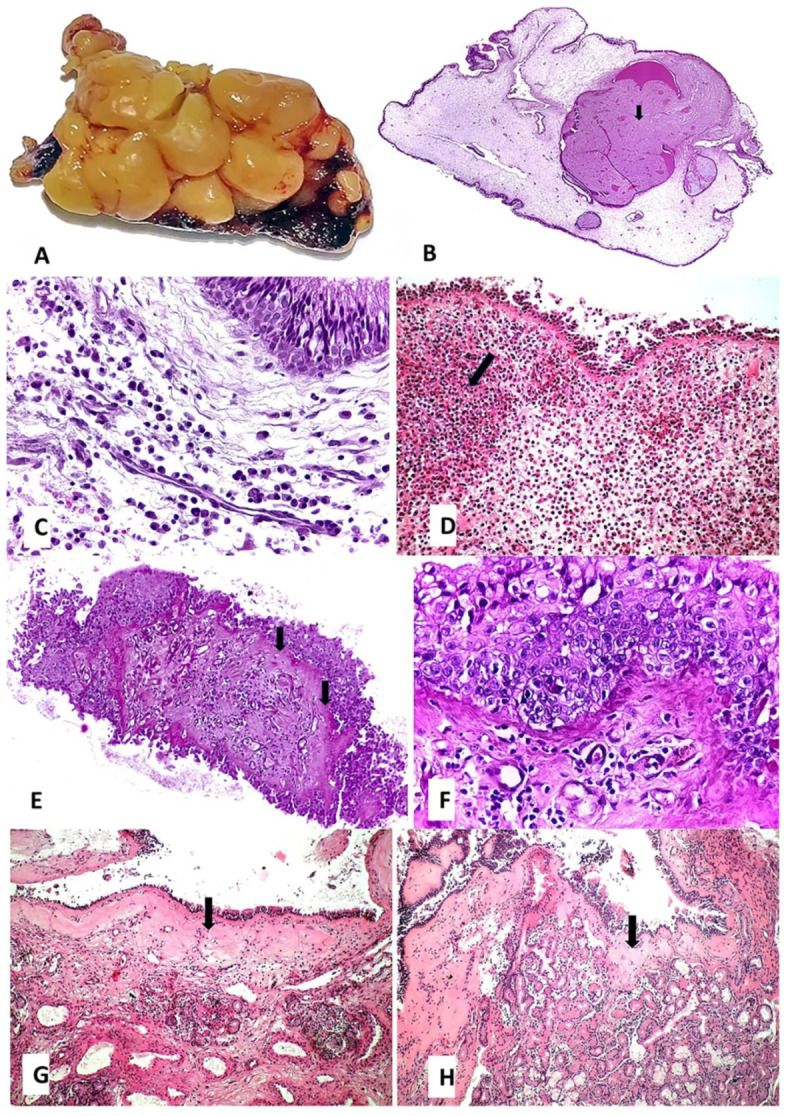

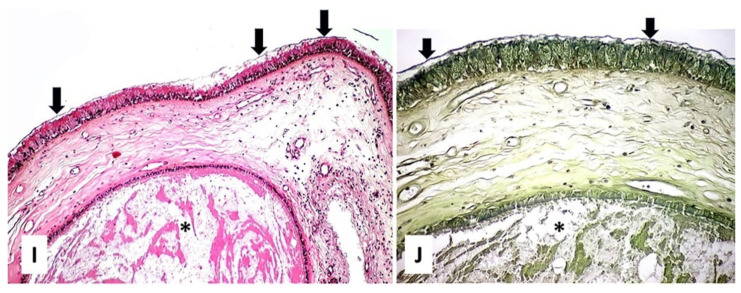
Pathology of CRSwNP: (**A**)—macroscopic aspect of edematous ethmoidal mucosa: edema generated a polypoid appearance (surgical resection fragment during FESS); (**B**)—interstitial edema with cystic mucus accumulation (mucocele) marked with arrow (HE ob. 4×); (**C**)—on the background of mucosal edema, focally, there is a mixed inflammatory infiltrate, consisting of mature lymphocytes mixed with plasma cells (HE ob. 40×); (**D**)—eosinophilic inflammatory infiltrate, cluster with eosinophils marked with arrow (HE ob. 20×); (**E**)—squamous metaplasia and thickening of the basal membrane (arrows) (HE ob. 4×); (**F**)—a detail of the squamous metaplasia shows no squamous layer and the presence of a hyperplastic basal and granular layer with some inflammatory cells migrated in the stratified epithelium (HE ob. 40×); (**G**)—basal membrane thickening (arrow) with some minimal inflammation in the lamina propria (HE ob. 10×); (**H**)—basal membrane thickening (arrow) with complete resolution of the inflammation (HE ob. 10×); (**I**)—chorionic fibrosis, biofilm on the surface epithelium (arrows), mucocele formation marked with * (HE ob. 40×); (**J**)—detail of the biofilm—bacteria covered by extracellular polysaccharide substance (arrows), mucocele formation marked with * (Gram ob. 40×).

**Figure 2:**
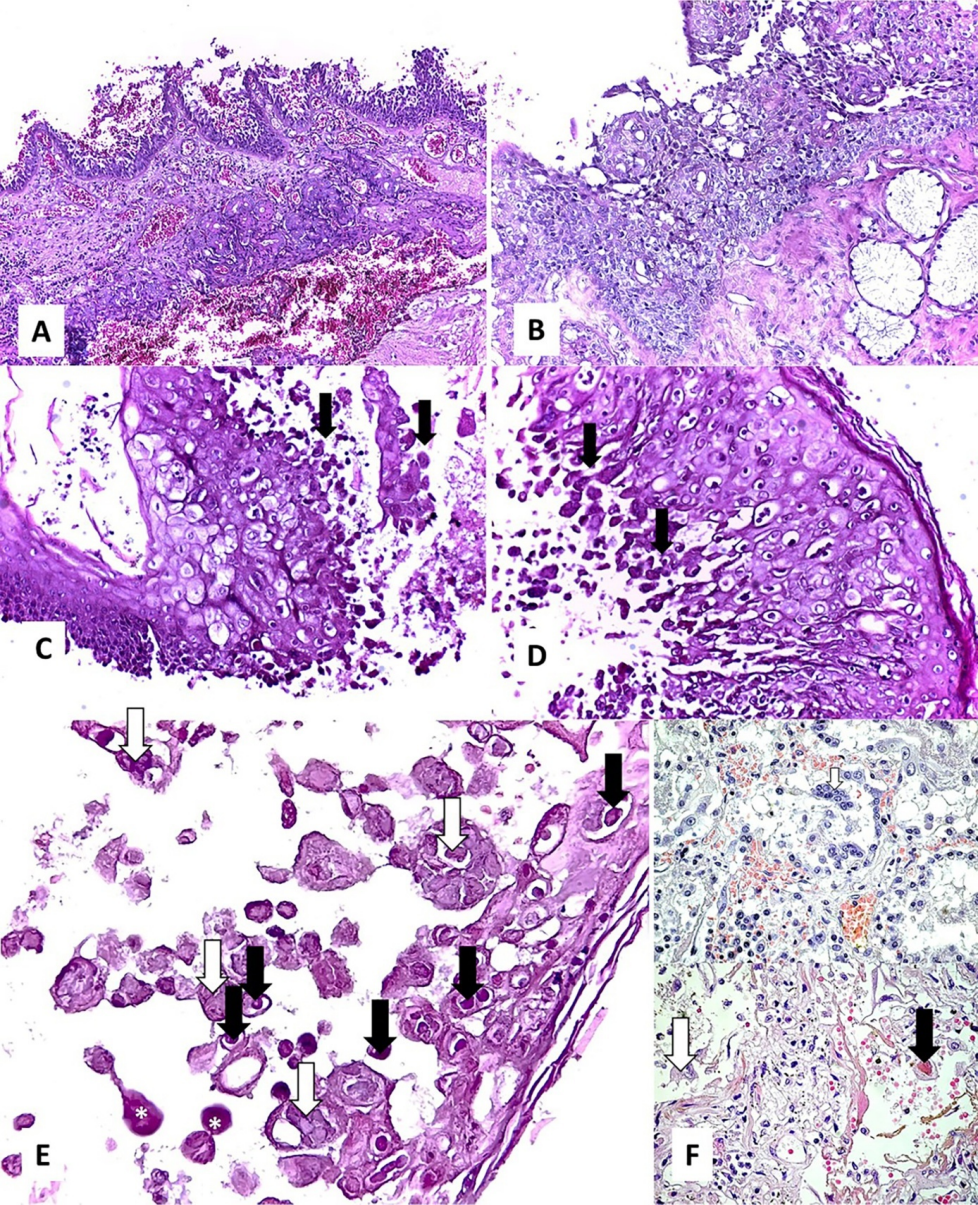
Histopathological findings in the nasal mucosa of COVID-19 patients: (**A**,**B**)—lymphocytic inflammation in the lamina propria, regardless of the absence ((**A**), HE ob. 10×) or presence ((**B**), HE ob. 20×) of squamous metaplasia; (**C**,**D**)—squamous metaplasia accompanied by a viral cytopathic effect in the hyperplastic basal layer (arrows) (HE ob. 20×); (**E**)—multinucleated giant cells (white arrows), prominent eosinophilic nuclear inclusion (black arrows) with cytomegaly, and some cells in induced necrobiosis (*) with nuclear lysis (HE ob. 40×); (**F**)—similar effect in alveolar macrophages in COVID-19 pneumonia (personal collection) (HE ob. 40×).

**Histopathology**. The histopathological aspects observed in the CRSwNP patients are presented in Figure 1, and those observed in the nasal mucosa of COVID-19 patients in Figure 2.

Stromal edema was found in 92% (*n* = 23/25) of the CRSwNP samples (Figure 1B), and only in 25% (*n* = 3/12) of the COVID-19 samples (statistically significant, *p* < 0.0001). While most of the CRSwNP samples with stromal edema showed mixed inflammation (24%, *n* = 6/25) (Figure 1C) and eosinophil-dominant infiltrate (56%, *n* = 14/25) (Figure 1D), in the COVID-19 samples with stromal edema, only lymphocyte-dominant infiltrate was observed (Figure 2A) (*p* = 0.3666). Squamous metaplasia (*p* = 0.4684), thickening of the basal membrane (*p* = 0.0768), and stromal fibrosis were detected in 28% (*n* = 7/25), 68% (*n* = 17/25), and 36% (*n* = 9/25) of the CRSwNP samples (Figure 1E–J), and in 41.6% (*n* = 5/12), 33.3% (*n* = 4/12), and 0 of COVID-19 samples (Figure 2B–D), respectively. In CRSwNP, basal membrane thickening was seen even after resolution of the process (Figure 1H), with or without restoration of normal ciliated respiratory epithelium.

Microbial biofilm was identified on the mucosal surface only in the CRSwNP group in 40% (*n* = 10/25) of the samples (Figure 1I,J).

In one case, viral cytopathic effect was identified in the hyperplastic basal layer (Figure 2C–E). This cytopathic effect consisted of the appearance of multinucleated giant cells, prominent eosinophilic nuclear inclusion with cytomegaly, and some cells in induced necrobiosis with nuclear lysis (Figure 2E). A somewhat similar effect was previously observed by us in alveolar macrophages in COVID-19 pneumonia as well (Figure 2F) (unpublished data).

**Figure 3:**
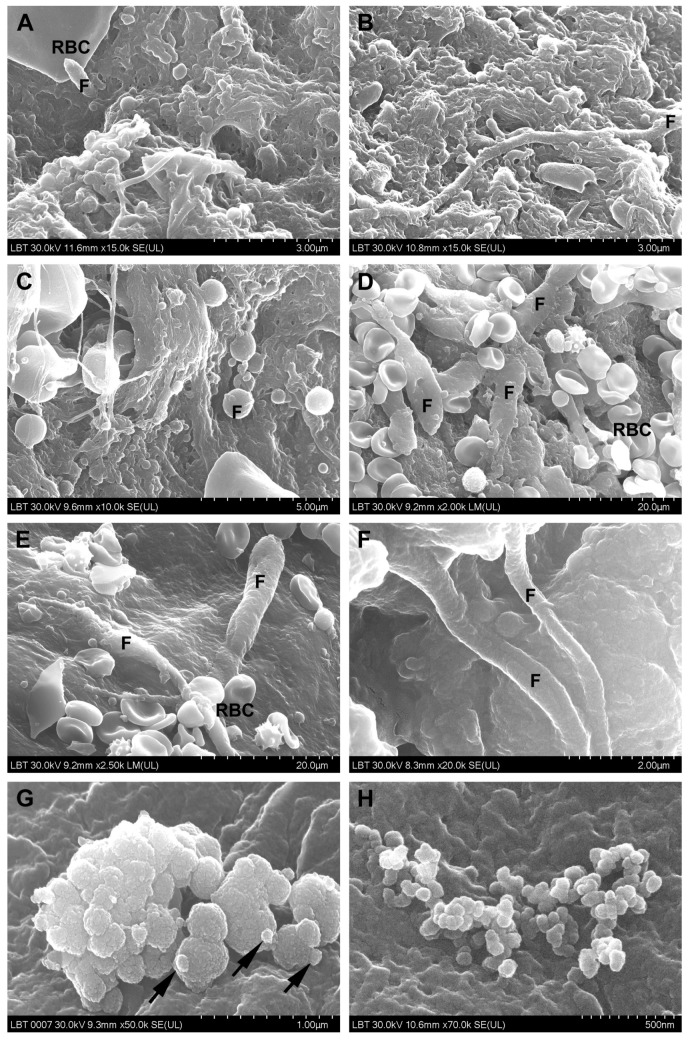
Surface of the nasal mucosa in CRSwNP samples (scanning electron microscopy): (**A**–**C**)—mixed microbial biofilms (bacteria and fungi); (**D**–**G**)—filamentous (**D**–**F**) and spherical fungal aggregates (**G**), the arrow indicates budding elements); (**H**)—nanomicrobial aggregate (F—fungi, RBC—red blood cell).

**Figure 4:**
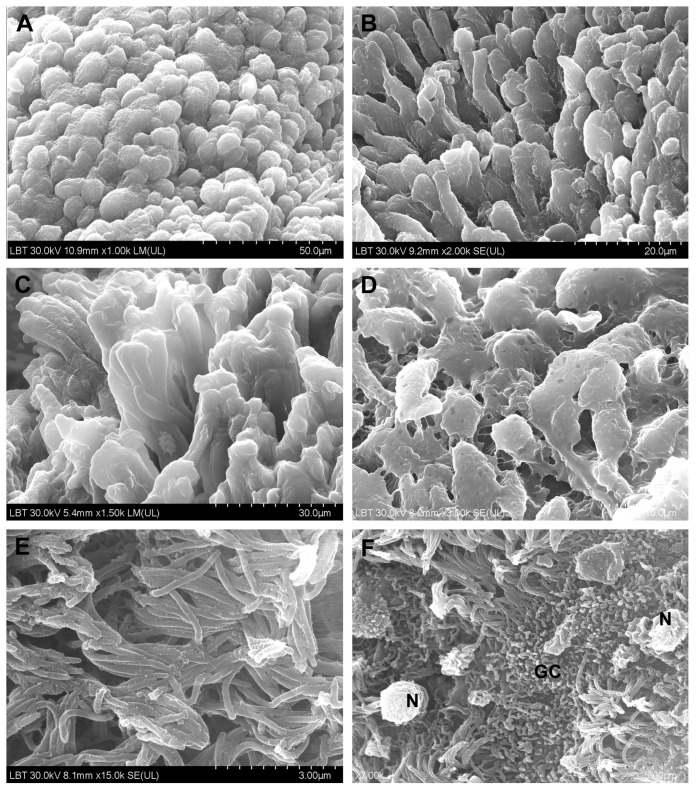
Microvilli and cilia in CRSwNP samples (scanning electron microscopy): (**A**,**B**)—microvilli with normal appearance; (**C**)—microvilli partially covered with mucus; (**D**)—mucus-embedded microvilli; (**E**)—cilia with normal appearance, mainly oriented in the same direction; (**F**)—ciliary disorientation, goblet cells area (N—neutrophil, GC—goblet cell).

**Figure 5:**
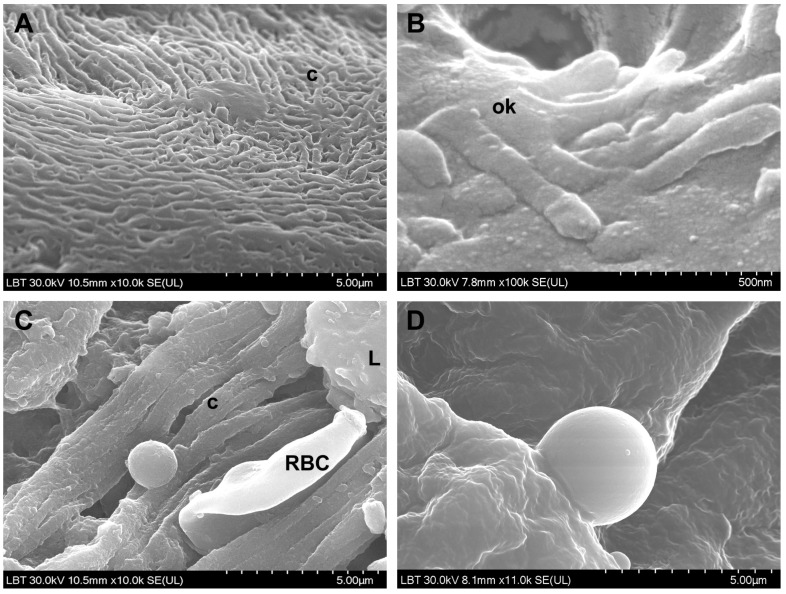
Surface of the nasal mucosa in control samples (scanning electron microscopy): (**A**)—cilia; (**B**)—olfactory cilia; (**C**)—rare microbial elements; (**D**)—smooth leukocytes (c—cilia, ok—olfactory knob, RBC—red blood cell, L—leukocyte).

**Figure 6:**
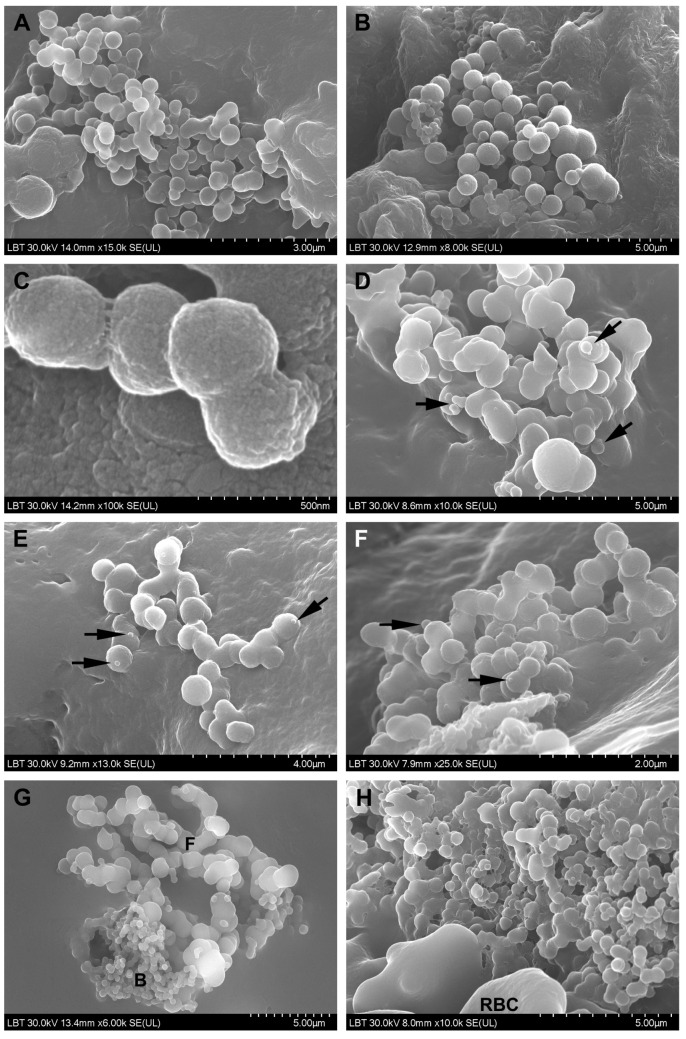

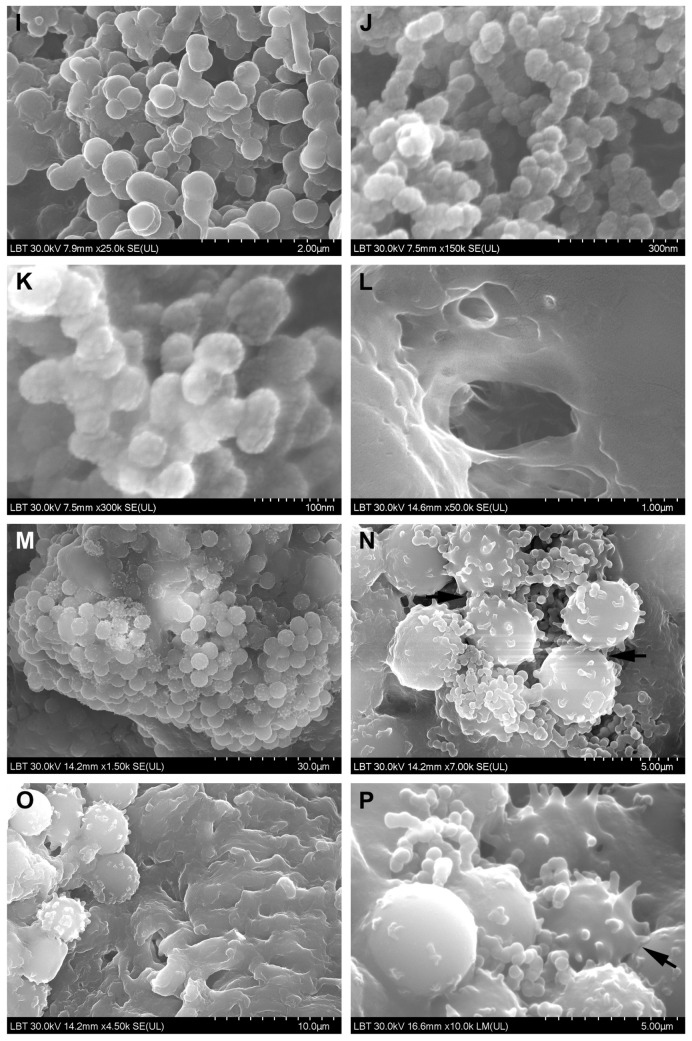

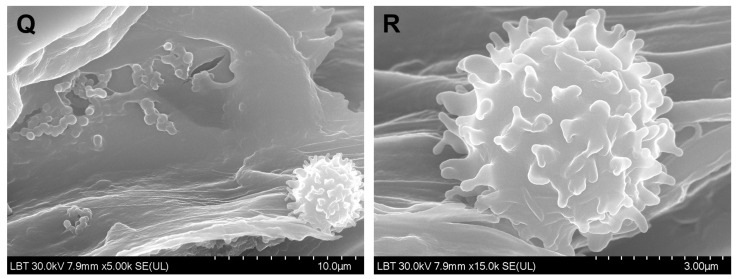
Surface of the nasal mucosa in COVID-19 samples (scanning electron microscopy): (**A**–**C**)—bacterial aggregates (cocci); (**D**–**F**)—fungal aggregates (the arrows indicate budding elements); (**G**–**I**)—mixed aggregates formed by bacteria and fungi; (**I**)—detail of (**H**); (**J**,**K**)—nanomicrobial aggregates; (**L**)—mucus without microbial elements; (**M**)—immune cell mass; (**N**)—detail of the preceding figure, which illustrates immunological synapses (arrows); (**O**–**Q**)—immune cells (the arrow indicates immunological synapses); (**R**)—detail of (**Q**); (F—fungi; B—bacteria; RBC—red blood cell).

**SEM**. The surface morphological aspects observed in CRSwNP samples are presented in Figure 3 and Figure 4, the aspects observed in the nasal mucosa of control patients in Figure 5, and those observed in the nasal mucosa of COVID-19 patients in Figure 6.

Microbial biofilms were identified only in CRSwNP samples (56%, *n* = 14/25) (Figure 3A–C), both bacterial and mixed (bacteria and fungi). Unlike the control group, where microbial elements (probably commensal bacteria) were detected in only one sample (20%, *n* = 1/5) in a relatively isolated manner (Figure 5C), most of the COVID-19 samples displayed surface-adherent microbial aggregates (statistically significant, *p* = 0.0148) that were not covered by extracellular polysaccharide substance (75% of the samples, *n* = 9/12) and were preponderantly bacterial (25%, *n* = 3/12, Figure 6A–C) or mixed (25%, *n* = 3/12, Figure 6G–I). Microbial aggregates were found in 28% of CRSwNP samples (*n* = 7/25), most of them fungal (12%, *n* = 3/25, Figure 3D–G). In both CRSwNP and COVID-19 the presence of nanomicrobial elements was detected (Figure 3H and Figure 6J,K).

We also identified cells belonging to the local immune system. These were observed in 58.3% (*n* = 7/12) of COVID-19 samples (Figure 6M–R) and in 28% (*n* = 7/25) of CRSwNP samples (*p* = 0.1452, Chi-square 3.860). Cell forming projections (Figure 6N,O,R), as well as cellular linkages (immunological synapses) (Figure 6N,P arrows) were observed. These cells were found to be in contact with bacteria (Figure 6M,N,P) or in close proximity (Figure 6Q).

Loss and dysfunction of cilia were seen in 76% of the CRSwNP samples (*n* = 19/25, Figure 4F) and in 33.3% of the COVID-19 samples (*n* = 4/12) (statistically significant, *p* = 0.0274).

**Figure 7:**
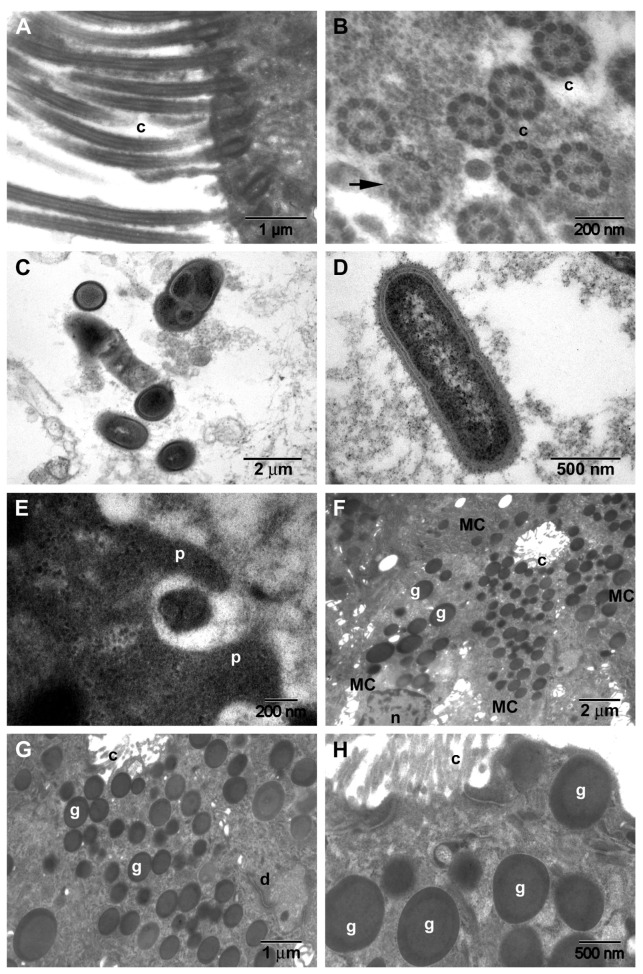

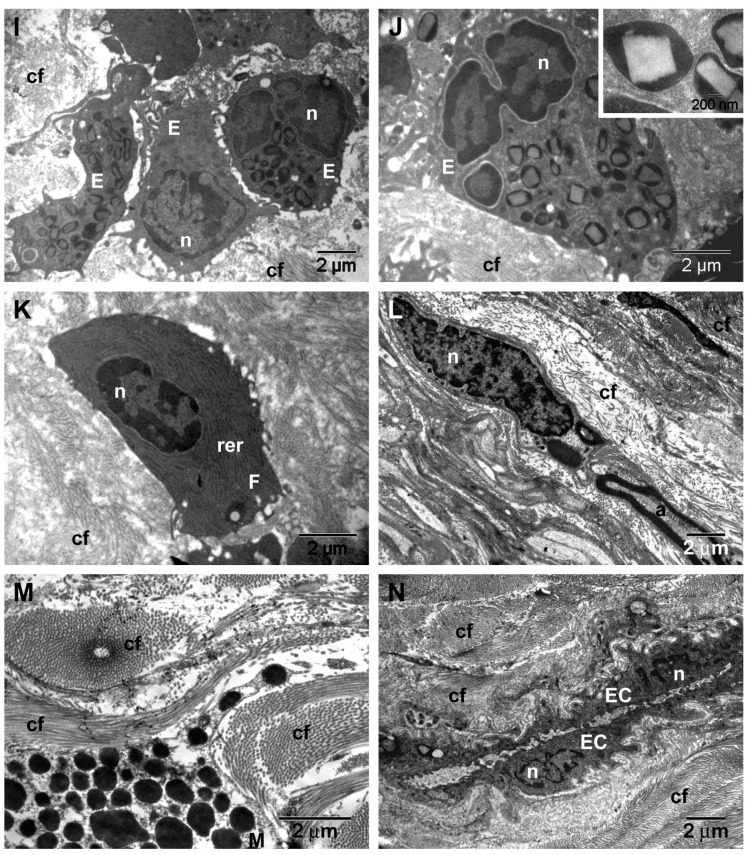
Transmission electron microscopy aspects of CRSwNP samples: (**A**)—normal cilia; (**B**)—normal cross-section of cilia and tubular anomalies (arrow); (**C**,**D**)—microbial biofilms; (**E**)—phagocytosis on the mucosal surface in the vicinity of the biofilm; (**F**–**H**)—mucous cell with abundant secretory granules ((**G**)—detail of (**F**)); (**I**,**J**)—eosinophils surrounded by collagen fibers; (**K**)—young fibroblast with intense protein metabolism; (**L**)—fibrocyte in the vicinity of an axon; (**M**)—mast cell surrounded by collagen fibers; (**N**)—capillary surrounded by collagen fiber bundles (subepithelial fibrosis) (c—cilia; p—pseudopod; g—mucus secretory granules; n—nucleus; d—desmosome; cf—collagen fibers; rer—rough endoplasmic reticulum; a—axon; MC—mucous cell; E—eosinophil; F—fibroblast; M—mast cell; EC—endothelial cell).

**Figure 8:**
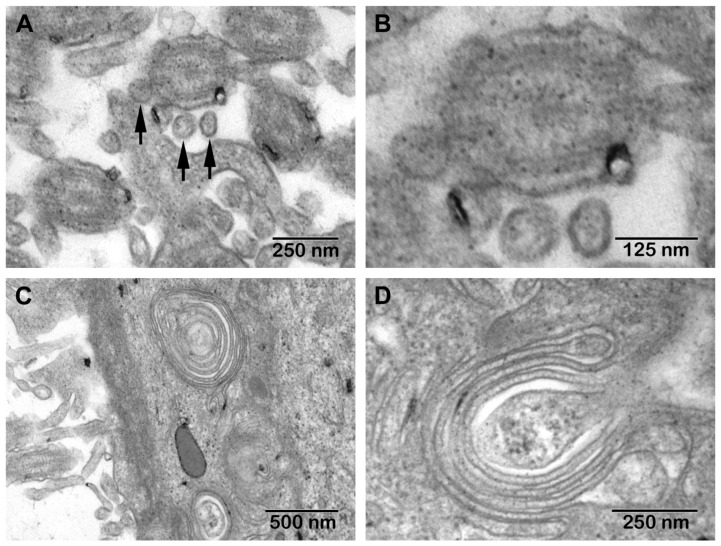
Transmission electron microscopy aspects of COVID-19 nasal mucosa: (**A**)—structures suggestive of the SARS-CoV-2 virus; (**B**)—detail of (**A**); (**C**,**D**)—multiple Golgi apparatus in epithelial cells.

**TEM**. The morphological aspects observed in CRSwNP are presented in Figure 7, and those seen in the nasal mucosa of COVID-19 patients in Figure 8.

In 39% (*n* = 14/25) of CRSwNP samples, surface microbial biofilms were identified (Figure 7C,D), in 44% (*n* = 11/25) ciliary abnormalities were detected (Figure 7B arrow) (*p* = 0.0581), and in 76% (*n* = 19/25) mucous cell hyperplasia was found (Figure 7F–H) (statistically significant, *p* = 0.0011). In the basal membrane and in the chorion, eosinophils (Figure 7I,J), fibroblasts (Figure 7K), fibrocytes (Figure 7L), and mast cells (Figure 7M) surrounded by collagen fiber bundles were observed (Figure 7N). In COVID-19 samples, in the extracellular area near the cilia, we identified the presence of structures suggestive of SARS-CoV-2 (enveloped particles with a double contour membrane and projections on the surface, and a heterogeneous, electron-dense, granular interior) (Figure 8A,B).

We observed both intact and disrupted respiratory epithelial surface areas. In most epithelial cells, multiple Golgi apparatus were observed (Figure 8C,D).

**Figure 9:**
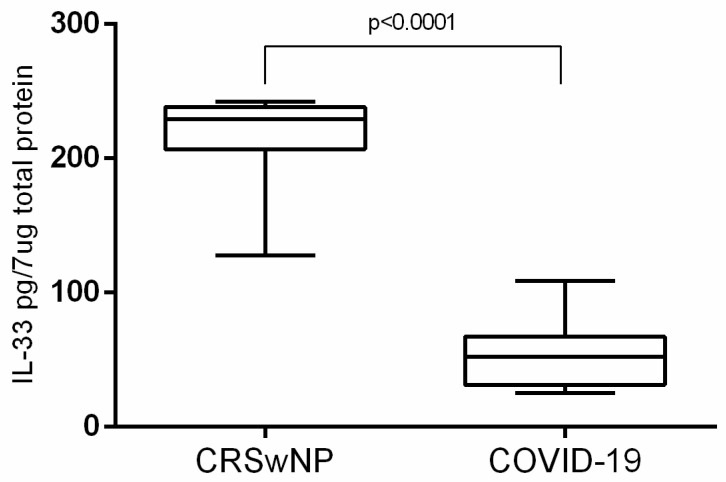
Tissue IL-33 concentration in the nasal mucosa (CRSwNP and COVID-19).

**IL-33**. The mean tissue IL-33 concentration in CRSwNP samples was statistically significantly higher than in COVID-19 samples (unpaired *t* test with Welch’s correction *p* < 0.0001). In CRSwNP samples, IL-33 had a mean tissue concentration of 210.0 pg/7 μg total protein (±8.327, *n* = 25) and in COVID-19 samples 52.77 pg/7 μg total protein (± 6.869, *n* = 12) (Figure 9).

**Table 2:** Age distribution, microscopic findings, and tissue IL-33 concentration of the nasal mucosa in CRSwNP versus COVID-19.

Age		COVID-19 Study	
	CRSwNP Study	COVID-19 Patients	Non-COVID-19 Patients (Control)
18–29	2	-	-
30–49	12	-	-
50–69	9	3	2
>70	2	9	3
**Total**	**25**	**12**	**5**
**Histopathology**			
Stromal edema (*p* < 0.0001)	92% (*n* = 23/25) (Figure 1B)	25% (*n* = 3/12)	-
- plasma cell-dominant	8% (*n* = 2/25)	-	-
- lymphocyte-dominant	12% (*n* = 3/25)	25% (*n* = 3/12) (Figure 2A,B)	-
- mixed inflammation	24% (*n* = 6/25) (Figure 1C)	-	-
- eosinophil-dominant	56% (*n* = 14/25) (Figure 1D)	-	-
- neutrophil-dominant	-	-	-
Squamous metaplasia	28% (*n* = 7/25) (Figure 1E,F)	41.6% (*n* = 5/12) (Figure 2B–D)	-
Basal membrane thickening	68% (*n* = 17/25) (Figure 1G,H)	33.3% (*n* = 4/12)	-
Stromal fibrosis	36% (*n* = 9/25) (Figure 1I,J)	-	-
Viral cytopathic effect	-	8.3% (*n* = 1/12) (Figure 2C–E)	-
Microbial biofilms	40% (*n* = 10/25) (Figure 1I,J)	-	-
**Scanning electron microscopy analysis**			
Microbial biofilms	56% (*n* = 14/25)	-	-
- bacterial	36% (*n* = 9/25)		
- mixed	20% (*n* = 5/25) (Figure 3A–C)		
Microbial aggregates (without biofilm) (*p* = 0.0148, Chi-square 8.422)	28% (*n* = 7/25)	75% (*n* = 9/12)	20% (*n* = 1/5)
- bacterial	4% (*n* = 1/25)	25% (*n* = 3/12) (Figure 6A–C)	20% (*n* = 1/5)
- fungal	12% (*n* = 3/25) (Figure 3D–G)	16.6% (*n* = 2/12) (Figure 6D–F)	-
- bacterial and fungal	4% (*n* = 1/25)	25% (*n* = 3/12) (Figure 6G–I)	-
- nanomicrobial	8% (*n* = 2/25) (Figure 3H)	8.4% (*n* = 1/12) (Figure 6J,K)	-
Surface immune cells	28% (*n* = 7/25) (Figure 4F)	58.3% (*n* = 7/12) (Figure 6M–R)	20% (*n* = 1/5) (Figure 5D)
Loss and dysfunction of cilia (*p* = 0.0274)	76% (*n* = 19/25) (Figure 4F)	33.3% (*n* = 4/12)	-
**Transmission electron microscopy analysis**			
Ciliary abnormalities	44% (*n* = 11/25) (Figure 7B)	8.3% (*n* = 1/12)	-
Microbial biofilms	36% (*n* = 9/25) (Figure 7C,D)	-	-
Hyperplasia of goblet cells (*p* = 0.0011)	76% (*n* = 19/25) (Figure 7F–H)	16.6% (*n* = 2/12)	-
**Assessing of tissue interleukin-33**			
	210.0 pg/7 μg total protein ± 8.327 (*n* = 25)	52.77 pg/7 μg total protein ± 6.869 (*n* = 12)	

## 4. Discussion

We chose to compare the alterations in the nasal mucosa in CRSwNP and COVID-19 because CRSwNP is one of the most studied rhinological disorders.

We noticed that the inflammatory pattern was different in the two conditions. The cytopathic effect already reported in the lungs of COVID-19 patients could be observed in the nasal mucosa of these patients. While biofilms were found on the mucosal surface of CRSwNP patients, in most COVID-19 samples we observed microbial aggregates indicating dysbiosis.

### 4.1. Microscopic Inflammatory Findings in CRSwNP

We identified in CRSwNP samples stromal edema with eosinophilic infiltrate, basement membrane thickening, and stromal fibrosis, but most of the COVID-19 samples did not show these changes.

Nasal polyps are inflammatory masses of the nasal mucosa (Figure 1A) that are covered with an intact respiratory epithelium. They have a thickened basal membrane under which stromal edema containing inflammatory infiltrate develops [20] (Figure 1B).

CRSwNP is characterized by a predominant type 2 inflammatory response mediated by T helper 2 cells [21]. Based on the dominant inflammatory cell types infiltrating the stroma, structured histopathology has identified several histotypes of CRSwNP (phenotypic clusters): plasma cell-dominant, lymphocyte-dominant, a mixed inflammation phenotype (Figure 1C), eosinophil-dominant (Figure 1D) and neutrophil-dominant [22]. These CRSwNP histotypes (Figure 1C,D) were also identified in our study, except the neutrophil-dominant one. The eosinophil-dominant phenotype is present in the majority of CRSwNP patients from United States and Europe and can be correlated with the Western lifestyle [23].

CRSwNP was classified into the eosinophil (ECRSwNP) subtype—a phenotype in which tissue eosinophils are predominant among inflammatory cells—and the non-eosinophil (nonECRSwNP) subtype [24]. However, a clear definition of ECRSwNP based on histopathological diagnostic criteria that can be applied uniformly has not yet been established [25]. ECRSwNP diagnosis can be biased by several processing factors (insufficient amount of polyp biopsy tissue, sectioning of the paraffin block) or interpretation factors (the microscopic fields selected by researchers, eosinophil numbers/high power field or eosinophil percentage) [26], which is why we used this classification in the current work research.

Goblet cell numbers in CRSwNP (Figure 7F–H) were found to be significantly higher than in the control group [27]. Our research showed that hyperplasia of goblet cells is statistically significantly much more frequently found in CRSwNP than in COVID-19 (76% vs. 16.6%) (Table 1). It seems that goblet cells play direct roles in the regulation of innate immunity by modulating immunological responses to infections and allergens [28].

Squamous metaplasia (Figure 1E,F), basal membrane thickening (Figure 1G,H), and stromal fibrosis (Figure 1I,J and Figure 7I–N) are morphological aspects of tissue remodeling secondary to chronic stromal edema [29]. With the onset of fibrosis, the number of inflammatory cells is reduced. The relationship between inflammation and tissue remodeling is a complex associative process, superior to a simple cause–effect relationship. A recent study [26] showed that stromal edema and basal membrane thickening are more frequent in CRSwNP patients than in CRSsNP patients, but no difference in stromal fibrosis between CRSwNP and CRSsNP groups was observed.

In the tissue remodeling process, the eosinophil plays a major, primary role [26] (Figure 7I,J). Patients with greater blood and tissue eosinophilia have a greater risk of recurrence and unsatisfactory results after FESS [30], probably because of histological tissue remodeling particularities.

Mast cells (Figure 7M) are important factors in allergic inflammation [31] and can influence the development of nasal polyps in CRS, being a significant source of Th2 cytokines [32]. Mast cell activation is IL-33-dependent [33]. The proportion of mast cells in the nasal mucosa of patients with CRSwNP was increased compared to patients with CRSsNP, regardless of atopic status [34].

Loss and dysfunction of cilia, observed also in our work (Figure 4F), are well known in CRS [35]. A recent study [36] maintained that mitochondrial damage may contribute to dysfunction in the beating of cilia in CRSwNP.

### 4.2. Microscopic Inflammatory Findings of the Nasal Mucosa in COVID-19 Patients

The inflammatory pattern in COVID-19 seems morphologically simpler than that found in CRSwNP. In the few cases where we observed stromal edema, it was lymphocyte-dominant (Figure 2A,B). Lymphocytic infiltration has been also observed in COVID-19 patients in other adjacent areas [37]. Changes described in the lower airways (trachea, bronchi, and bronchioles) also include CD3 T-lymphocyte infiltrate and thickening of the mucosa [38]. The squamous metaplasia and basal membrane thickening we found in COVID-19 patients were, most probably, preexistent, as the result of a chronic respiratory aggression (smoking or other pollutants).

The appearance of a cytopathic viral effect like that observed by us in the nasal mucosa (Figure 2C–E) has been reported in the lungs in other viral respiratory infections [39], as well as in COVID-19 [40]. The lack or diminution of inflammatory reaction suggests a more probable apoptosis mechanism of necrobiosis. Apoptosis was observed in human airway epithelial cell cultures infected with SARS-CoV-2 [41].

Inoculation of healthy volunteers with human coronavirus caused disruption of the ciliated epithelium and ciliary dyskinesia [42]. In our research, loss and dysfunction of cilia were found in 33.3% (*n* = 4/12) of COVID-19 samples.

A high genomic load was identified in most COVID-19 mucosal samples. Respiratory tract viral loads increased the risk of death [43]. Zou et al. showed that viral load increases after symptom onset, with higher viral loads detected in the nose than in the oral cavity [44]. In a previous study of deceased patients we found that the viral load in the mucosa of the middle ear was lower than the nasal mucosa (deceased patients). [45].

### 4.3. Microbial Surface Communities

Microbial communities on the surface of the nasal mucosa can be commensal, symbiotic, or pathogenic microorganisms. The distinction between the commensal and pathogenic flora is frequently ambiguous; some microorganisms can be both commensal and opportunistically pathogenic [46]. In one of our previous studies [47], we identified several opportunistic agents in the etiology of CRS in immunocompetent patients.

Nasal dysbiosis plays an important pathogenetic role in the development of respiratory and otic diseases, also identified in COVID-19 [48]. SARS-CoV-2-induced nasal dysbiosis, evidenced by us through the increase in bacterial and fungal colonization, can be due to a direct viral effect on the microbiota (similar to other viruses [49]), as well as to a secondary effect through the deterioration of the epithelial barrier [42], making it susceptible to the subsequent invasion by other pathogenic or opportunistic agents. Thus, COVID-19-associated coinfections were correlated with mechanical ventilation [50] and broad-spectrum antimicrobial therapy [51,52]. Our electron microscopic results reinforced the idea of nasal dysbiosis in severely ill COVID-19 patients. To our knowledge, this is the first article that evaluated by SEM the aspect of the nasal mucosa in deceased COVID-19 patients.

Bacterial and fungal biofilms are frequently present in patients undergoing FESS for CRSwNP (where they are even more prevalent as compared to other forms of CRS), but biofilms are also present in controls without CRS [2,52,53]. Thus, it seems that the presence of biofilms is not sufficient to cause CRS without other host cofactors [54]. The biofilm detection rates vary depending on the type and the working method used. Conventional SEM sample preparation methods do not always preserve the structure of the extracellular polysaccharide substance [55], making image interpretation difficult. Also, the biofilm may be discontinuous and may not be detected on the collected fragments. The cryofixation SEM variant provides better preservation of extracellular polymeric substances [56].

Unfortunately, TEM has several disadvantages: it involves a much more cumbersome working technique, it focuses on a very small area at the expense of the overall biofilm, and obtaining a sufficiently thin sample may affect the biofilm [57], which is why TEM has a lower biofilm detection rate (in our research 36%, *n* = 9/25 vs. SEM 56%, *n* = 14/25).

Fungal aggregates are very frequently identified in the mucus of the nasal cavity and paranasal sinuses, in both CRSwNP patients [58] and healthy individuals. Thus, the simple presence of fungal aggregates is not a diagnostic criterion. The relationship between fungi and eosinophilic inflammation is not clearly understood, but fungi may induce the recruitment, activation, and degranulation of eosinophils [59], and progression toward the disease or not, depending on host factors.

The smallest microbial elements we identified by SEM on the surface of the nasal mucosa in both CRSwNP (Figure 3H) and COVID-19 (Figure 6J,K) patients and in control samples have a round shape and sizes of 50–100 nm. From a morphological point of view, their classification as ultramicrobacteria (which we tend to support) or viruses can be discussed. A recent study [60] isolated filtrable ultramicrobacteria 200–400 nm in size from the nose, throat, and skin of pediatric patients. The role and implication of ultramicrobacteria in pathogenetic processes are not known. Additionally, identifying viruses in the biofilm remains a technically and analytically difficult task, so the role of viruses in CRS is almost unknown. It is known that viral biofilms might constitute a key reservoir for chronic infections [61,62].

Coronaviruses can be confused in TEM samples with normal cell organelles, and autolysis of cells can complicate morphological assessment; SARS-CoV-2 TEM characteristics can orient identification (enveloped particles with double contour membrane and projections on the surface, a heterogeneous, electron-dense, partly granular interior; intracellular particles are typically located within membrane compartments [63,64,65,66]). TEM characteristics of SARS-CoV-2 can be negatively affected by autolysis of cells, complicating cell type assessment [66]. We identified particles suggestive of extracellular SARS-CoV-2 in the area between cilia.

Coronaviruses are assembled by budding at the interface between the endoplasmic reticulum and the Golgi apparatus (intermediate compartment). The absence of coronaviruses from Golgi stacks suggests that these leave the cells through an unconventional pathway [67].

### 4.4. Surface Immune Cell Communities

Certainly, there is a complex crosstalk between immune cells residing in mucosal compartments: dendritic cells and macrophages are sentinel cells for the invading agents, T cells attack and eliminate pathogenic agents, and B cells secrete IgA [68,69]. The nasal mucosa also contains a dense network of professional antigen-presenting cells, in both the epithelium and the lamina propria comprising macrophages and various subsets of immature dendritic cells [70].

The cells observed by us (Figure 7M–R) were immature dendritic cells or lymphocytes, or both categories of cells. Dendritic cells in peripheral tissues have an immature phenotype, an increased phagocytic capacity (for capturing the antigen), and a reduced antigen-presenting capacity [71]. After absorbing the antigen, these dendritic cells move from the mucosa to locoregional lymph nodes, where they mature and initiate adaptive immune responses. Physical interactions (immunological synapses) between professional antigen-presenting cells and resident T cells have also been identified in the nasal mucosa, indicating constant local immunological control [70]. Dendritic cells are certainly implicated in the pathogenesis of COVID-19, but the mechanisms are not yet known [72]. An increase in the number of mature dendritic cells in the bronchoalveolar lavage of COVID-19 patients has been reported, suggesting that these cells are involved in pulmonary immune response secondary to SARS-CoV-2 infection [73].

Morphologically, immature dendritic cells have a 6–9 μm diameter, a round shape and a smoother surface, while mature cells have 10–15 μm diameters, a rough surface with multiple pseudopods—long cytoplasmic extensions, known as dendrites [74,75,76], sometimes difficult to identify by SEM. Lymphocytes have a heterogeneous phenotype; they vary in size, but most of them are small, with a 6–9 µm diameter, and they show on their surface varying numbers of stubby or finger-like microvilli. Morphologically, T and B lymphocytes cannot be accurately differentiated [77,78]. In addition, T cells show various morphological alterations (elongation-flattening-rounding) during immunological synapse with dendritic cells [79].

**IL-33.** The fact that IL-33 was found in a higher concentration in the nasal mucosa of CRSwNP patients than in COVID-19 patients can be explained by the long-term chronic aggression of CRSwNP and by the different inflammatory model. In any case, this insight should be interpreted with caution because no previous studies have reported similar results when assessing tissue IL-33 in deceased patients. It is not known how tissue IL-33 concentration can be influenced post-mortem.

IL-33 functions as an alarmin molecule that is released from cells following various lesions with the aim of alerting immune cells that express the ST2 receptor (also known as IL-1RL1), leading to activation of the NF-κB pathway in various innate and adaptive immune cells. It is not clear whether IL-33 is secreted by activated immune cells or is directly released because of cell death [11]. Mature IL-33 boosts type 2 immunity via the activation of group 2 innate lymphoid cells, eosinophils, mast cells, macrophages, and T helper 2 cells [9,80].

In CRSwNP, IL-33 mediates eosinophilic infiltration, induces mucus production and goblet cell hyperplasia [81], and is involved in mucosal edema, subepithelial collagen deposition, and infiltration of neutrophils [11]. Several studies have shown that IL-33 mRNA and IL-33 protein levels in the CRSwNP group are significantly higher and ST2, the ligand-binding chain of the IL-33 receptor, is elevated [3,11,81,82,83,84].

Unlike oligosymptomatic or asymptomatic patients, in critically ill COVID-19 patients, higher plasma cytokine levels were identified [85] and correlated with patient survival [86]. The role of IL-33 in COVID-19 is unknown, but it is speculated that IL-33 might even play a key role in driving all stages of this disease [13], including the progression to healing or hyperinflammation and thromboses [12]. In bronchoalveolar lavage fluid from patients with mild to severe COVID-19, a population of IL-33-producing cells, which increases with the disease, was identified [87]. Cell line studies showed that SARS-CoV-2 infection promotes IL-33 expression in human epithelial cells [13]. High plasma IL-33 levels in severe COVID-19 infection might result from lesions of the lower respiratory cells, caused by the interaction between respiratory epithelium and activated immune cells [13]. Serum ST2 levels were persistently high in non-surviving severe cases [88].

Research has shown that after the resolution of the COVID-19 infection, convalescent individuals have persisting peripheral blood mononuclear cells that produce IL-33 in response to virus-specific T cell activation, in correlation with seropositivity. IL-33 production is correlated with CD4+ T cell activation, which is most probably because of the T cell-mediated effects on IL-33-producing cells [87].

There are limitations to the current research, such as the small number of cases in both studied groups, which influenced the statistical significance of results. The lack of immunohistochemical determinations limited the interpretation of results, especially regarding lymphocytes involved in COVID-19 inflammation. Morphological changes were classified dichotomously as present or absent; further stratification might allow additional results and interpretations. Additionally, IL-33 is only a secondary pathway of CRS.

Our research included only severely ill patients with both diseases. Therefore, the results can not be extrapolated to other severity groups. 

Yet our results may serve as a starting point for other studies, such as research on nasal dysbiosis and its effects, especially in critical patients, the potential role of probiotics in this context, or larger studies on IL-33 at different tissue levels.

## 5. Conclusions

Although the two disorders may share common morphological characteristics, the existing inflammatory patterns are different. Results have suggested the presence of nasal dysbiosis in both conditions, which can be a determining factor in CRSwNP and a secondary factor in COVID-19.

The nasal mucosa of deceased COVID-19 patients presents multiple microbial aggregates and the intense implication of surface immune cells also undergoing cytopathic viral effects. Studying the initial host–virus interaction in the nasal microbiota can be one of the ways to understand the appearance and modulation of systemic inflammatory response in COVID-19. Given that pulmonary viral seeding is secondary to nasal seeding, further rhinological research in COVID-19 is required for studying local factors that initiate systemic hyperinflammatory responses, as well as investigating the possibility of developing an intranasal vaccine.

The nasal mucosa of CRSwNP patients is also characterized by dysbiosis. A study of the interaction among the etiological factors of chronic inflammation in CRSwNP will bring us closer to an optimal individualized treatment of this disorder, particularly in refractory cases.

Studying the role of probiotics in the modulation of the nasal microbiota will benefit future studies.

## Figures and Tables

**Table 1:** Research design.

	CRSwNP Study	COVID-19 Study	
		Case Group	Control Group
Number of patients	25	12	5
Medical institution of the patients	2nd Otorhinolaryngology Clinic, University Clinical Hospital of Railway Company, Cluj-Napoca, Romania	Department of Pathology, County Emergency Hospital Deva/Institute of Legal Medicine Cluj-Napoca	Department of Pathology, County Emergency Hospital Deva/Institute of Legal Medicine Cluj-Napoca
Inclusion criteria	Patients undergoing functional endoscopic sinus surgery for CRSwNP, diagnosed according to the EPOS 2020 criteria [4]	Deceased patients with antemortem COVID-19 diagnosis confirmed through combined throat/nasal sampling RT-PCR SARS-CoV-2 test	Deceased patients, with antemortem negative RT-PCR SARS-CoV-2 test performed through combined throat/nasal sampling
Exclusion criteria	- age under 18 years - positive RT-PCR SARS-CoV-2 test from combined throat/nasal sampling - secondary causes of CRS - previous rhinosinusal surgery - under topical antibiotic/corticosteroid treatment less than three weeks before operation - anterior nasal trauma - sino-nasal malignancy - radiation therapy to the head and neck - septal perforation - autoimmune diseases, sarcoidosis, granulomatosis of the nasal cavity, diabetes mellitus - pregnant women - cystic fibrosis, Kartagener syndrome - insufficient samples	- age under 18 years - nasogastric intubation - known rhinosinusal pathologies - malignant ENT tumors - known autoimmune diseases - pregnant women - cystic fibrosis, Kartagener syndrome - insufficient samples	- age under 18 years - antemortem positive RT-PCR SARS-CoV-2 test from combined throat/nasal sampling - nasogastric intubation - known rhinosinusal pathologies - ENT malignant tumors - known autoimmune diseases - pregnant women - cystic fibrosis, Kartagener syndrome - insufficient samples
Research analysis	Histopathology, electron microscopy analysis, and assessing of tissue interleukin-33	Tissue RT-PCR SARS-CoV-2, histopathology, electron microscopy analysis, and assessing of tissue interleukin-33	Tissue RT-PCR SARS-CoV-2, histopathology, and electron microscopy analysis

## Data Availability

The virology analysis results are available at the Department of Cell and Molecular Biology, Iuliu Hatieganu University of Medicine and Pharmacy, Cluj-Napoca, Romania; hmatei@umfcluj.ro (H.V.M.). The results of histopathologic and electron microscopy exams are available at the Department of Anatomy and Embryology, Iuliu Hatieganu University of Medicine and Pharmacy, 400006 Cluj-Napoca, Romania; Contact: jeican.ionut@umfcluj.ro.
